# Uremic Toxins in Organ Crosstalk

**DOI:** 10.3389/fmed.2021.592602

**Published:** 2021-04-16

**Authors:** Jerome Lowenstein, Sanjay K. Nigam

**Affiliations:** ^1^Department of Nephrology, New York University School of Medicine, New York, NY, United States; ^2^Departments of Pediatrics and Medicine (Nephrology), San Diego School of Medicine, University of California, San Diego, La Jolla, CA, United States

**Keywords:** indoxyl sulfate, aryl hydrocarbon receptor, organ crosstalk, uremia, OAT knockout

## Abstract

Many putative uremic toxins—like indoxyl sulfate, p-cresol sulfate, kynurenic acid, uric acid, and CMPF—are organic anions. Both inter-organ and inter-organismal communication are involved. For example, the gut microbiome is the main source of indole, which, after modification by liver drug metabolizing enzymes (DMEs), becomes indoxyl sulfate. Various organic anion transporters (organic anion transporters, OATs; organic anion-transporting polypeptides, OATPs; multidrug resistance-associated proteins, MRPs, and other ABC transporters like ABCG2)—often termed “drug transporters”—mediate movement of uremic toxins through cells and organs. In the kidney proximal tubule, critical roles for OAT1 and OAT3 in regulating levels of protein-bound uremic toxins have been established using knock-out mice. OATs are important in maintaining residual tubular function in chronic kidney disease (CKD); as CKD progresses, intestinal transporters like ABCG2, which extrude urate and other organic anions into the gut lumen, seem to help restore homeostasis. Uremic toxins like indoxyl sulfate also regulate signaling and metabolism, potentially affecting gene expression in extra-renal tissues as well as the kidney. Focusing on the history and evolving story of indoxyl sulfate, we discuss how uremic toxins appear to be part of an extensive “remote sensing and signaling” network—involving so-called drug transporters and drug metabolizing enzymes which modulate metabolism and signaling. This systems biology view of uremic toxins is leading to a new appreciation of uremia as partly due to disordered remote sensing and signaling mechanisms–resulting from, and causing, aberrant inter-organ (e.g., gut-liver- kidney-CNS) and inter-organismal (e.g., gut microbiome-host) communication.

## Introduction

There is a new appreciation of the role of the kidney in organ cross-talk and inter- organismal communication (e.g., gut microbiome-host) mediated by small organic molecules. Many of these molecules are organic anions transported by renal (e.g., OAT1) and non-renal transporters (OATP1B1) and altered by Phase 1 and Phase 2 drug metabolizing enzymes (DMEs); many of these molecules also have well-described roles in metabolism, signaling, and modulation of redox state (1). Among these are so-called protein-bound uremic toxins (e.g., indoxyl sulfate, kynurenate, CMPF, phenyl sulfate, p-cresol sulfate, uric acid), which accumulate in chronic kidney disease (CKD) as tubular secretion declines. One of the most fascinating aspects of current research in the field is the multifaceted nature of these uremic toxins and their roles in “remote sensing and signaling” between organs and organisms. Here, we detail a few examples from a systems biology perspective with an emphasis on how they illustrate aspects of the Remote Sensing and Signaling Theory.

Before discussing the role of uremic toxins in organ crosstalk, it is worth outlining several key concepts of the Remote Sensing and Signaling Theory (RSST) relevant to CKD. The RSST is a general systems biology theory of how transporters and enzymes help optimize remote communication by small organic molecules between organs (e.g., gut-liver-kidney-brain) and organisms [e.g., gut microbes-host; (1)].

At least 500 transporters, enzymes, and regulatory proteins (e.g., nuclear receptors and other transcription factors) participate in this remote sensing and signaling (RSS) system. Many of the proteins in the RSS system have a broad substrate specificity and are well-known in the pharmaceutical field for their involvement in the absorption, distribution, metabolism and excretion (ADME) of drugs such as NSAIDs, statins, antibiotics and antivirals. Thus, certain SLC and ABC transporters and enzymes in the RSS system are often referred to as drug transporters and drug metabolizing enzymes (DMEs).

However, while well-known for their pharmaceutical roles, homologs of many of the genes encoding these proteins are found in mice, fish, flies, and other organisms. This strongly suggests an important role for these proteins in the physiology of very diverse organisms. Furthermore, it is now clear from *in vitro* and *in vivo* studies in model organisms as well as human genome wide association studies (GWAS) that the proteins of the RSS system interact with a wide range of metabolites, signaling molecules, antioxidants, nutrients, and gut microbe products. These small molecules participate in key physiological and biochemical pathways, including those involving bile acids, the citric acid cycle, fatty acid oxidation, cellular redox state, and a wide variety of signaling events.

It is through the movement, modification, and action of these small organic molecules between organs and body fluids—as well as between organisms—that remote sensing and signaling is believed to occur. Thus, the transporters, enzymes, and regulatory proteins of the RSS system—a network of over 500 proteins differentially expressed across tissue and cell type—help maintain homeostasis and also help restore homeostasis in the context of injury to the kidney and other organs. Among the small molecules regulated by the RSS system are many protein-bound uremic solutes and uremic toxins accumulating in CKD that are taken up by the proximal tubule. One of the best-studied of these uremic toxins is indoxyl sulfate.

## The Many Faces of Indoxyl Sulfate

Although indoxyl sulfate has only become broadly known to nephrologists in recent decades as a small, protein-bound, likely uremic toxin, in fact, indoxyl sulfate—and the related *indigo* and *indican*—have a long and rich history. Indoxyl sulfate is a small organic molecule—an organic anion—that is considered to be one of the most important protein-bound uremic toxins originating in the gut. Early in the twentieth century the indigo-related molecule, indoxyl sulfate, was recognized as a solute capable of binding to plasma albumin (2). It was observed that plasma concentrations of indoxyl sulfate were increased in patients with reduced renal function, as judged by elevated serum creatinine concentration, and in a 5/6 nephrectomy rat model, administration of indoxyl sulfate in the diet or by gavage resulted in tubular and glomerular damage and hastened the development of uremia (3). Studies such as these led to the designation of indoxyl sulfate as a *uremic toxin* rather than a protein-bound uremic solute.

It is interesting to briefly contemplate the parallels, real, and analogical, between the binding of indoxyl sulfate to plasma proteins and the use of indigo—one of the most valued dyes from antiquity, a tight-binding dye applied to fabrics. This tight binding of indican or indoxyl sulfate to plasma proteins was of considerable interest to pharmacologists and physiologists (2). Pharmacologists have long recognized that many drugs (and colored dyes used as biological probes) are protein bound, and that binding is an important determinant of active (unbound) drug concentrations and drug removal. It was recognized that there was competition among solutes for binding to albumin, and some solutes bound more tightly than others. Indoxyl sulfate was shown to be 95% protein-bound in normal plasma and to compete, *in vitro*, for binding with other colored dyes for binding in uremic plasma (2).

Protein-binding is physiologically important because binding limits removal of solutes by glomerular filtration. Protein-bound solutes such as indoxyl sulfate, an organic anion, are transported from peritubular capillaries into the renal tubule largely by two major organic anion transporters (OATs) on the basolateral surface of proximal renal tubular cells (4). In progressive renal disease, indoxyl sulfate concentration in plasma increases as nephron (tubular) mass declines. Lacking transporters, removal by dialysis is limited by the low concentration of free (diffusible) solute. Although it was recognized that protein-binding would impede removal by glomerular filtration or removal across a dialysis membrane, the possible role of indoxyl sulfate as a uremic toxin initially received little attention. In early experiences with the membrane dialyzer, it was recognized that small molecules were removed much more readily than indoxyl sulfate. However, the long-term consequences of the limited removal of indoxyl sulfate and other protein-bound uremic solutes and uremic toxins were not evident in the early days in which hemodialysis was limited to one or two treatments (5).

Interest in the importance of “protein-bound uremic solutes” was stimulated when it became clear that patients with end-stage renal disease (ESRD) exhibited a high propensity to cardiac death during the first 3 years of hemodialysis (6). Most of the deaths were attributed to heart failure, cardiac arrhythmias, or “sudden death.” Variation in hemodialysis techniques and dialysis membranes aimed at more efficient removal of urea and creatinine–taken as surrogates for other dialyzable uremic toxins—did not have a significant effect on this excess mortality (7). Although this was generally attributed to coincident risk factors (hypertension, diabetes, hyperlipidemia, and cigarette smoking) in the ESRD population, a high incidence of cardiovascular disease was reported in dialysis patients with few conventional risk factors for cardiovascular disease (8).

During the period dating from 1960 through the early 2000's when chronic renal failure was treated by thrice-weekly hemodialysis, only modest attention was given to the nature of the toxins removed by hemodialysis (9). After almost 50 years, during which the population of patients receiving hemodialysis in the US swelled, nephrologists finally turned their attention to the protein-bound solutes retained in patients receiving standard hemodialysis. In 2003, the EUTox group, based on a thorough analysis of the literature, identified and categorized more than 100 solutes that were reported to be increased in concentration in the serum of patients with impaired renal function. Direct measurements largely confirmed this identification of uremic solutes (10). In the context of a growing interest in trying to improve dialysis outcomes, as well as attempting to understand the pathobiology of uremia in molecular terms, attention began to focus on small solutes rendered poorly-dialyzable by virtue of protein binding. The possibility that some of the group of poorly-dialyzed protein-bound solutes that accumulated in the blood of patients with advanced renal disease were responsible for the excess morbidity and mortality was implied but not established. Accordingly, the group of solutes identified by the EUTox group were termed either *uremic toxins* or *uremic retention solutes*.

**Figure 1:**
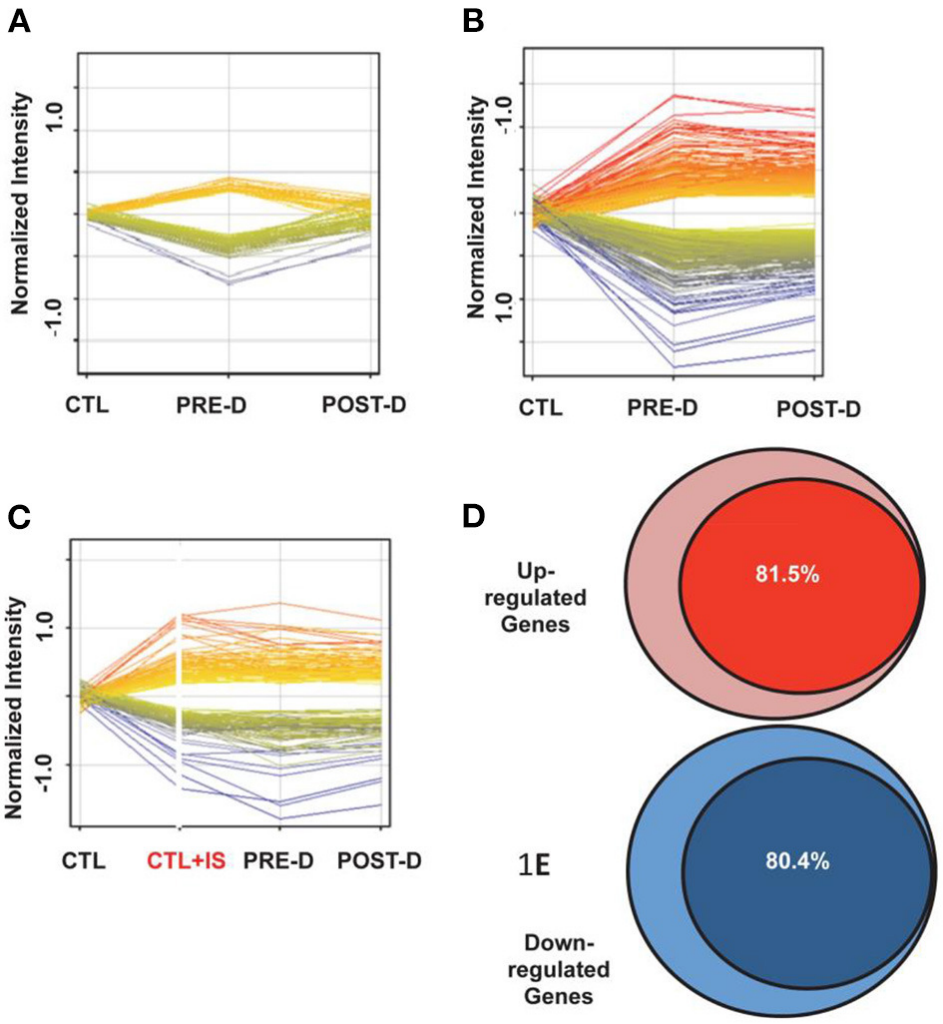
Gene expression in reporter renal tubular cells. **(A)** Gene expression changes that returned to baseline post-dialysis. Each individual line represents the average value in the 10 uremic subjects for each gene that was dysregulated. The expression of 282 genes was upregulated (displayed as yellow to red lines) and the expression of 255 genes was downregulated (displayed as green to blue lines) in the pre-dialysis samples as compared to normal controls. Post-dialysis these values returned to baseline. **(B)** Gene expression that remained dysregulated after dialysis treatment. The expression of 843 genes was upregulated and the expression of 532 genes were downregulated. **(C)** Genes expressed following incubation in normal plasma spiked with indoxyl sulfate compared with their expression levels in cells treated with pre-dialysis and post-dialysis plasma. The expression of 908 genes was upregulated and the expression of 571 genes were downregulated. **(D)** 81.5% of upregulated genes that were not normalized by dialysis were mimicked by addition of indoxyl sulfate to normal plasma. **(E)** 80.4% of downregulated genes that were not normalized by dialysis were mimicked by addition of indoxyl sulfate to normal plasma.

Indoxyl sulfate is currently among the most widely discussed of uremic toxins and among the best studied in mechanistic terms. Indoxyl sulfate is but one of many on the list. Others include p-cresol sulfate, kynurenine, TMAO (trimethylamine oxide), polyamines, and CMPF (3-carboxy-4-methyl-5-propyl-2-furanpropanoic acid). Although some are organic cations (e.g., TMAO), a large number are, like indoxyl sulfate, small organic anions. In recent years, several lines of evidence have led to the view that indoxyl sulfate and some other uremic solutes function as *signaling molecules* as well as potential toxins. An effect of uremic plasma on gene expression was demonstrated in normal human renal tubular cells (11). In human renal tubular cells incubated for 24 h with control and pre- and post-dialysis uremic plasma, the expression of more than 2,000 genes was increased or decreased. These changes, in roughly 500 genes, were reversed when cells were incubated in post-dialysis plasma (Figure 1A) consistent with the fact that many retention solutes, such as urea and creatinine, are freely dialyzable.

However, more than 1,500 genes remained dysregulated when cells were incubated with post-dialysis plasma (Figure 1B), consistent with the conclusion that the stimulus for gene expression was probably protein-bound and therefore not readily dialyzable. Further, it was observed that spiking control plasma with indoxyl sulfate to a concentration typical of that observed in patients with ESRD, simulated roughly 80% of the dysregulation observed with uremic plasma (Figures 1C–E). The effects of indoxyl sulfate on gene expression with uremic plasma or control plasma spiked with indoxyl sulfate were blocked by probenecid, an inhibitor of organic anion transport. These findings suggested strongly that indoxyl sulfate activates a transcriptional program dependent upon one or more organic anion transporters.

## Uremic Toxins and the Renal Organic Anion Transporters, OAT1 and OAT3

In addition to recent interest in signaling mechanisms involving indoxyl sulfate, there is renewed interest in the transport of uremic toxins like indoxyl sulfate by organic anion transporters (OATs) and other transporters in the proximal tubule in chronic kidney disease (CKD). Renal transport not only regulates the elimination of uremic toxins but also plays an important role in setting the systemic and proximal tubule cell levels of these small organic molecules. In a sense, this renewed attention on proximal tubule handling of solutes has led to the “rediscovery” of renal tubule microperfusion studies performed in the 1970's (12, 13). Grantham observed that isolated rabbit renal proximal tubules could *secrete* fluid. It was argued that secretion of fluid must have been preceded by the transport of some solute into the tubular lumen that created an osmotic driving force. Grantham observed that addition of p-aminohippurate, which is now known to be taken up by OATs in the proximal tubule, to the bathing medium stimulated fluid transport. Further, it was observed that plasma from ESRD patients, added to the bathing medium, caused fluid *secretion* in perfused rabbit renal tubules, which in retrospect, suggests that some molecule(s) in uremic serum acted as a “signal” to induce tubular secretion of fluid. Much of the “signal” was most likely the osmotic effect of solute transported into the tubular lumen. But with a growing appreciation of the importance of these small organic molecules in signaling, it is possible that specific signaling mechanisms within the tubular cells also help explain the phenomenon. Nevertheless, these older studies suggested that, despite poor glomerular filtration, or possibly because of it, the OATs in the proximal tubule contribute to the elimination of indoxyl sulfate and other uremic solutes. As Grantham wrote, “under conditions of markedly reduced or complete cessation of glomerular filtration, mammalian proximal tubules could secrete fluid and hippurate and similar substances… elimination of some of the potentially toxic products normally excreted by the kidneys… would serve a useful survival function” (13). In this regard, it is interesting to note reports that proximal renal tubular secretion of indoxyl sulfate is increased in animals manifesting reduced overall renal function. Recent studies reported that increasing the production and serum concentration of indoxyl sulfate by protein-feeding resulted in the upregulation of OAT gene expression in isolated renal tubular cells and epithelial cells isolated from human urine (14). This might account for the observation that renal tubular secretion of indoxyl sulfate is increased in patients manifesting “residual renal function” (15, 16). Importantly, these patients have been observed to have a better quality of life, and longer survival independent of the intensity of hemodialysis or peritoneal dialysis (17).

These findings have focused on the role of OATs in the renal tubule handling of indoxyl sulfate. OATs appear to be the main *in vivo* transporters of indoxyl sulfate and other protein-bound uremic toxins (18, 19). This has been shown by metabolomics studies in OAT knockout mice (4, 20). It is important to note, however, that OAT1 and OAT3 knockout mice, while lacking the main route for elimination of indoxyl sulfate and many other “toxins,” have normal life expectancy, despite many fold increase in some uremic toxins. In light of this, it is worth noting that they also have high levels of potentially beneficial metabolites, including those with antioxidant activity, which may be protective.

Furthermore, these uremic toxins have likely been elevated since birth and are known to affect tissue remodeling, which could conceivably be adaptive. Although indoxyl sulfate is one of the most altered uremic toxins in the OAT knockout mice, many other uremic toxins—mostly gut microbe-derived—are also elevated in the plasma of these knockout mice (4, 20). These other uremic solutes/uremic toxins include many indole derivatives, kynurenine, CMPF, p-cresol sulfate, phenyl sulfate, p-cresol sulfate, TMAO, urate, and other molecules that accumulate in uremic serum (21, 22). That OAT1 (originally called NKT for Novel Kidney Transporter) and/or OAT3 directly interact with many of these molecules has (with the possible exception of TMAO), been confirmed using *in vitro* cell- based transport assays. Thus, this *in vitro* data supports the *in vivo* genetic evidence (4) that OATs are part of a gut microbe-gut-liver-kidney axis (23).

**Figure 2:**
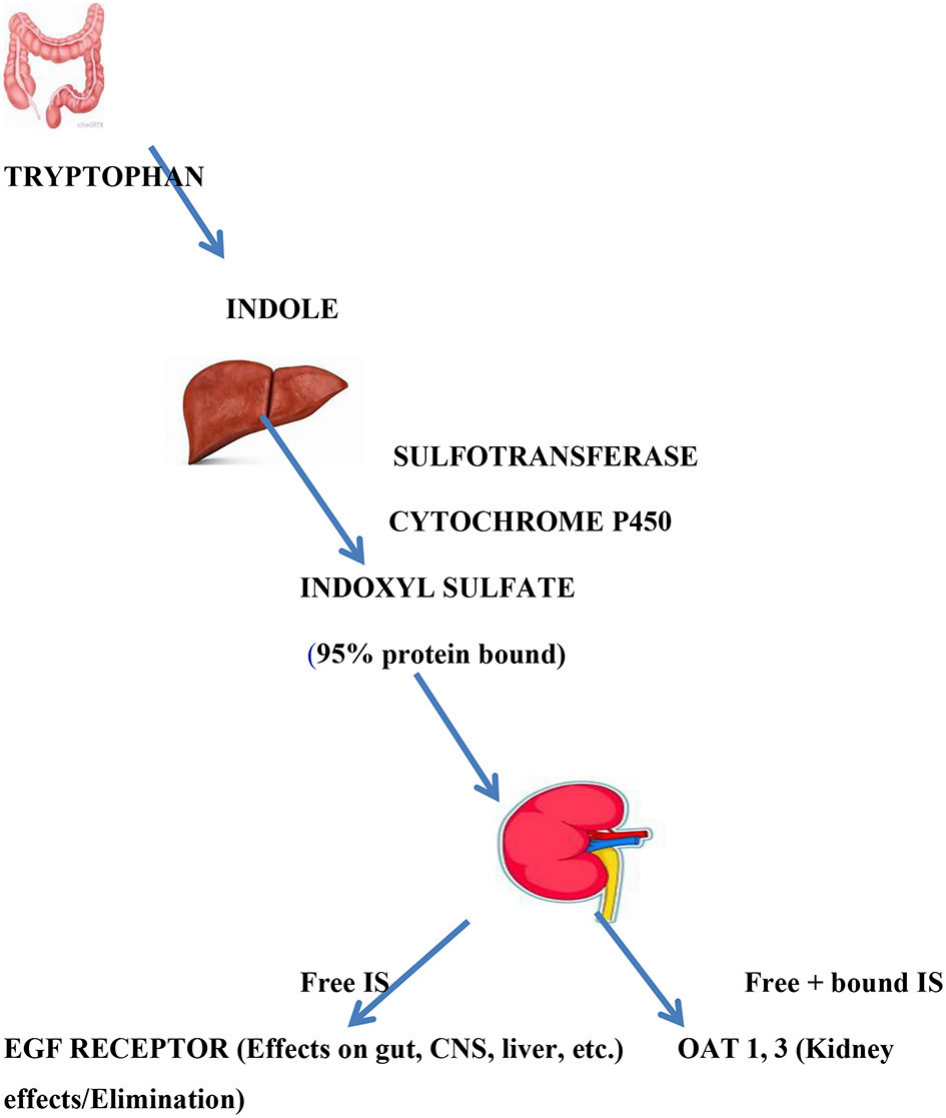
Indoxyl sulfate production and actions. Indoxyl sulfate is the product of gut bacterial tryptophanase and hepatic hydroxylation and sulfation. It is 95% albumin bound. Renal excretion, which serves to remove bound and free IS, is mediated by OAT 1 and OAT 3 in proximal renal tubule where signaling events and elimination occur. Free (unbound) IS can act as a signaling molecule on distant tissues bearing EGF receptors.

Indoxyl sulfate is known to be derived from the breakdown of tryptophan to indole by gut bacteria that express tryptophanase (24, 25). The product, indole, passes into the hepatic portal system, where it undergoes hydroxylation (by cytochrome p450) and sulfation (by a sulfotransferase) before it enters the hepatic vein and the general circulation as indoxyl sulfate. In the kidney, indoxyl sulfate is transported across the basolateral membrane of proximal tubule by OAT 1 and 3; on the apical membrane it is likely eliminated via MRPs (ABCC family) and other transporters (26). Thus, multiple “drug” metabolizing enzymes work together with multiple transporters to produce indoxyl sulfate and allow it to enter the proximal tubule cell whereupon it is eliminated. Again, we emphasize the roles of these transporters and DMEs in mediating inter-organismal (e.g., gut microbes-human) and inter-organ (e.g., gut-liver-kidney) “remote” communication affecting the plasma and tissue levels of indoxyl sulfate (Figure 2).

## Indoxyl Sulfate Signaling and the Remote Sensing and Signaling Theory

Homer Smith, a student of the evolution of the kidney (27), asked why so much fluid is filtered to yield a relatively small quantity of urine. The question seems to point to some kind of paradox with the appearance of a futile process. Smith answered by pointing out that glomerular filtration evolved when life moved into fresh water and the capacity to excrete fluid became a priority. A question of a similar sort might be asked.

“Are indoxyl sulfate and other uremic toxins made by a series of biochemical reactions only to be excreted?” The answer to this question, while far from fully resolved, is turning out to be quite interesting. Indoxyl sulfate and several other gut-derived protein- bound solutes are highly bound (>90%) in plasma. Renal excretion is facilitated by OATs on the basolateral (blood side) of proximal tubular cells that shift the equilibrium toward free solute that is readily transported into the tubular lumen. In this way, renal tubular secretion serves to remove suspect uremic toxins. A recent study (14) demonstrated, in intact mice and isolated human urinary epithelial cells, that increasing the plasma concentration of indoxyl sulfate by feeding protein resulted in a substantial increase in OAT1 synthesis and indoxyl sulfate excretion. When renal mass is reduced by renal disease, indoxyl sulfate concentration in plasma increases and enhanced transport of indoxyl sulfate into the proximal tubule by OAT1 and possibly OAT3 provides an important means for removing this potentially toxic solute. But this description of the role of OATs in the elimination of indoxyl sulfate and other anions might not have satisfied Homer Smith. He might have asked whether it was reasonable to believe that indoxyl sulfate was synthesized only to be secreted by the kidney.

This hypothetical question provides glimpses into a whole new line of systems level thinking that challenges current thinking about uremic toxins. The capacity of indoxyl sulfate to affect a wide range of targets follows in large part from evidence that it can be transported across the plasma membrane and interact with aryl hydrocarbon receptors (AHR) in the cytoplasm (28). The complex is stabilized by binding heat-shock proteins of the HSP90 family and passes through a pore into the nucleus where it releases the heat-shock proteins and combines with the Aryl Receptor Nuclear Translocator (ARNT). This binds to the Xenobiotic Response Element (XRE) and activates transcription of a wide range of gene products. Though generally termed a “*xenobiotic* response element (XRE)”—implying that it functions to inactivate *foreign* molecules–it has become increasingly apparent that binding of the AHR-ARNT to the “xenobiotic response element” also mediates responses to *endogenous* stimuli presented through binding to the aryl hydrocarbon receptor. Indeed, it seems likely that the XRE, which was originally perhaps best known for mediating the response to *exogenous* 2,3,7,8-tetrachlorodibenzo-*p*-dioxin (TCDD) exposure, has responded to small *endogenous* solutes long before “modern society” produced TCDD as a side product in organic synthesis and burning of organic materials. The ARNT complex leads to the expression of “drug” metabolizing enzymes (DMEs), SLC transporters, and ABC transporters. Other nuclear receptors, such as PXR (pregnane X receptor) and HNF4a (hepatocyte nuclear factor 4 alpha) (29) also regulate the expression of drug transporters and drug metabolizing enzymes in the kidney proximal tubule, liver, intestine, and many other organs.

A number of studies have now identified AHR as an important target of indoxyl sulfate. Indoxyl sulfate, bound to AHR, can activate a great number of genes, including cytochrome P450 enzymes, as well as genes involved macrophage-dependent inflammation (30). Today, the aryl hydrocarbon receptor (AHR) is probably better characterized as a ligand-activated transcription factor that functions in the integration of environmental, dietary, microbial and metabolic cues by regulating transcriptional programs. The nature of this regulation appears to be ligand-dependent, cell-type-specific, and context-specific. Along these lines, it is worth considering the cells of the proximal tubule in this way—as well as specific metabolites and signaling molecules transported into the proximal tubule cells that might affect the activation of AHR and/or other nuclear receptors.

Recent data indicates that free (unbound) indoxyl sulfate (and other aryl hydrocarbons) can bind to the EGF receptor of cells lacking OATs and induce a signaling cascade that results in ARNT translocation and initiation of gene transcription (31). This proximal tubule signaling role of indoxyl sulfate is consistent with the Remote Sensing and Signaling Theory as applied to the context of CKD. The Remote Sensing and Signaling Theory describes how a large network of genes—differentially expressed in the gut, liver, kidney as well as other organs and traditionally viewed as central to the absorption, distribution, metabolism, and elimination (ADME) of drugs—regulates inter-organ and inter-organismal communication mediated by small organic molecules of “high informational content.” Among these small organic molecules with high informational content are included rate-limiting metabolites (e.g., carnitine, TCA intermediates), signaling molecules (e.g., short chain fatty acids, prostaglandins), and antioxidants (e.g., uric acid, ergothionine). To this list of molecules with high informational content one can now add certain uremic solutes and uremic toxins.

So, the answer to the hypothetical question Homer Smith might have posed is that, while the renal clearance by OATs and other transporters serves to prevent excessive buildup of uremic toxins, unbound uremic solutes may be important signaling molecules, metabolites, and antioxidants acting upon remote organs (e.g., brain) or remote organisms (e.g., gut microbes, nursing infant; Figure 2).

## Altered Remote Sensing and Signaling in CKD

**Figure 3:**
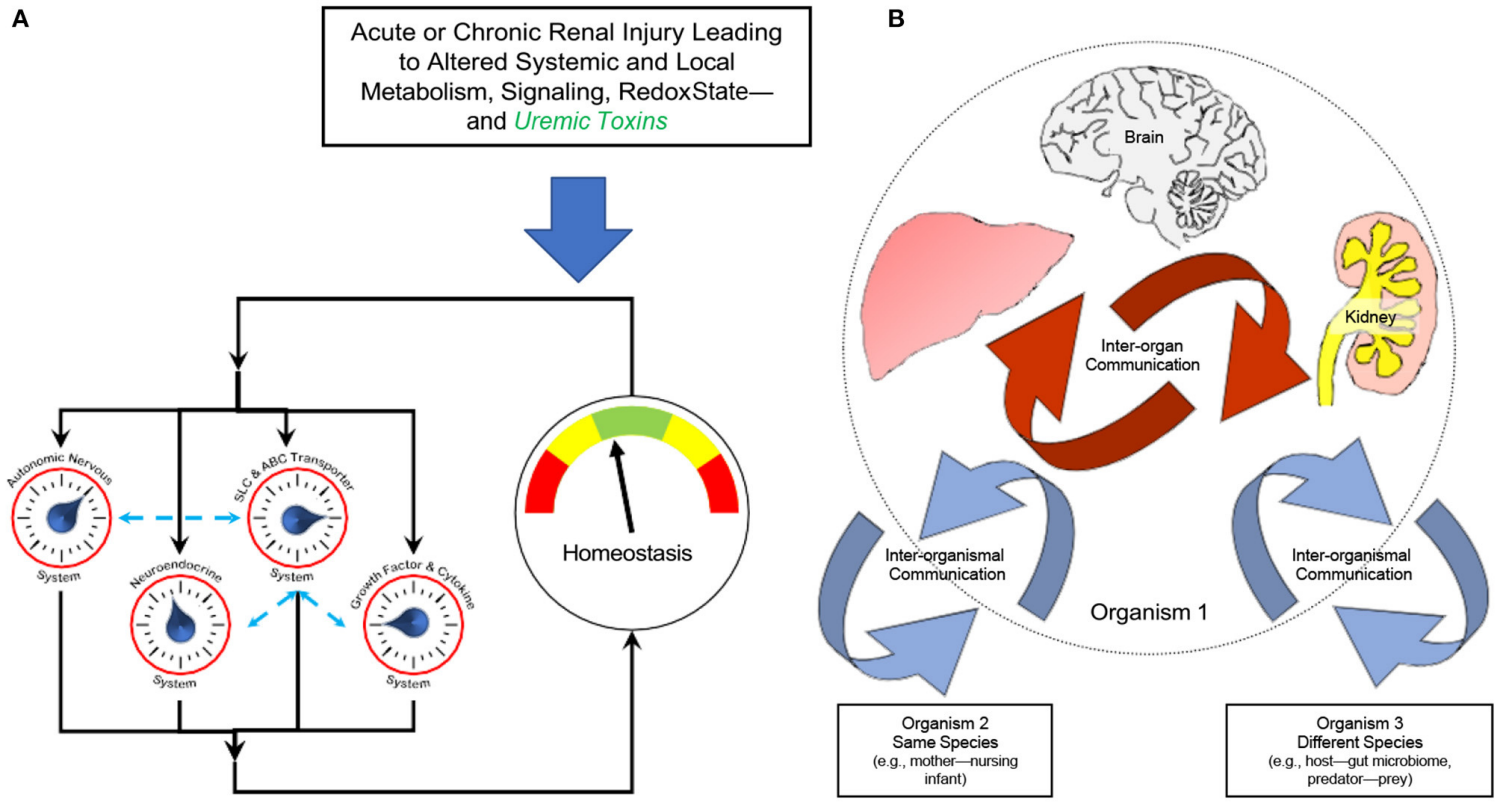
Altered remote sensing and signaling in the context of kidney disease and uremic toxins. The Remote Sensing and Signaling Theory describes how proteins often considered to be primarily involved in the absorption, distribution, metabolism, and excretion (ADME) of small molecule drugs—including multispecific “drug” transporters like renal OAT1 and Phase 2 “drug” metabolizing enzymes like liver sulfotransferases—actually form a network mediating endogenous small molecule remote communication between organs and organisms. These small molecules have “high informational content” in that, like indoxyl sulfate, they are central to metabolism, signaling, and regulation of oxidative state. The expression of genes in the multiorgan (e.g., gut-liver-kidney) remote sensing and signaling network is regulated by, among other factors, nuclear receptors such as AHR, HNF4a, and FXR. A number of multi-specific transporters and enzymes work together with those of more limited (oligo- and mono-) specificity to potentially handle tens of thousands of endogenous and exogenous molecules in health and disease (e.g., CKD). **(A)** In acute or chronic organ injury, this complex adaptive system has the ability to reset until endogenous small molecule homeostasis is partially or completely restored. The Remote Sensing and Signaling System works in concert with other homeostatic systems (e.g., neuroendocrine system). **(B)** Inter-organismal communication can be between organisms of the same species (e.g., mother-nursing infant) or different species (e.g., gut microbes-host). Based on (1, 32, 34, 44).

It is generally held that chronic kidney disease (CKD) and uremia are complex syndromes involving altered inter-organ and inter-organismal remote communication affecting metabolism, signaling, and redox potential—among many other biochemical and cellular processes. In the past decade, indoxyl sulfate has been the uremic toxin at or near the center of research in this field, and the mechanism of its action as a signaling molecule in the proximal tubule is even more complex than we have described— involving not only OAT1 and AHR, but also EGF receptor, oxidative state, and a microRNA (31). Furthermore, it is increasingly believed that, like indoxyl sulfate, many other uremic toxins act as both signaling molecules and are also the cause of many of the deleterious manifestations of uremia (Figure 3).

Indoxyl sulfate is a good example of the importance of small molecule inter-organ and inter-organismal (e.g., gut microbes-host) communication in the body in that, as described above, *in vivo* it follows the gut microbe-gut-liver-kidney axis and requires the participation of “hepatic drug metabolizing enzymes” (Phase 1 for hydroxylation and Phase 2 for sulfation) and drug “transporters” like the renal OATs (30). In other words, indoxyl sulfate is one of perhaps thousands of signaling molecules and metabolites regulated by the Remote Sensing and Signaling System described above. The network (and subnetworks) of ADME genes regulating this system's effort at optimizing small molecule levels in various tissues and body fluid compartments consists of multi- specific “drug” transporters, “drug” metabolizing enzymes, transcription factors,—and other transporters (oligospecific, monospecific), enzymes, and regulatory proteins. A preliminary Remote Sensing and Signaling Network has recently been built for the gut-liver-kidney axis (32). Many of the molecules that are optimized—such as metabolites and signaling molecules—are themselves ligands for receptors central to endogenous physiology (e.g., G-protein coupled receptors, nuclear receptors). This transporter- and DME-based Remote Sensing and Signaling System is also intimately connected to more classical homeostatic systems (e.g., neuroendocrine, autonomic), and like them, appears critical for helping the organism restore homeostasis after perturbation, whether acute or chronic (e.g., increased indoxyl sulfate concentration in chronic kidney disease).

Although the Remote Sensing and Signaling Theory was originally formulated over a decade ago to synthesize a growing amount of data related to the biological role of “drug” transporters that did not conform to the view that their primary role was in drug absorption, distribution, metabolism, and excretion (ADME), the applicability of the RSST to CKD and uremia has become increasingly apparent (33–36). A small sampling important of results are: (1) the fact that gene expression data has revealed changes in “drug” transporters and “drug” metabolizing enzymes in non-renal organs (e.g., intestine, liver) that appear to compensate for the inability of the injured kidney to transport small molecules like urate and indoxyl sulfate, (2) the realization that many protein-bound uremic toxins such as indoxyl sulfate are among the best substrates of “drug” transporters like the OATs, (3) an appreciation of the importance of inter- organismal communication between the gut-microbes and the host, the former being the ultimate source of many of the best-known uremic toxins.

With the intense recent attention given to the mechanisms by which uremic toxins and solutes affect cell and organ function, there is a rapidly growing appreciation of CKD and uremia as a multi-organ disorder involving altered metabolism and signaling mediated by small organic molecules—implying the involvement of specific metabolic and signaling pathways definable at the cellular and molecular levels–as well as a renewed appreciation of the importance of residual renal function which in large part represents the activity of proximal tubule transporters of protein-bound molecules (e.g., OAT1).

Given the emphasis in the Remote Sensing and Signaling Theory on the role of multi-specific transporters and enzymes, as well as their regulators (e.g., nuclear receptors) in remote inter-organ and inter-organismal small molecule communication (including gut microbe-host), the theory serves as a basis for understanding disordered small molecule communication in disease settings like CKD and uremia (35). In this context, it is worth mentioning that, in the setting of progressive renal disease, the predominantly intestinal multi-specific ABC transporter ABCG2 (BCRP) appears to play an important role in helping to restore human uric acid homeostasis by extruding it, and possibly other uremic toxins, into the intestinal lumen (36). This has been interpreted as an example of remote sensing and signaling in humans. Potential mechanisms include effects of uric acid or uremic toxins like indoxyl sulfate on ABCG2 expression or function in intestinal epithelial cells (37, 38).

The growing understanding of the mechanistic basis of indoxyl sulfate action serves as a good example of the need to think beyond toxins and examine effects upon specific signaling and metabolic pathways. While nephrologists have been conditioned to think in terms of uremic “toxins,” this traditional view is challenged by a number of observations. First, most uremic toxins and uremic solutes are present in the body in the absence of kidney dysfunction. In addition to the OATs, there are transporters of these small molecules in many non-renal tissues. Crucially, some of these solutes, such as kynurenine and polyamines, are known to participate in major biological pathways independent of kidney disease. One possibility, consistent with a growing amount of biochemical and molecular data, is that so-called uremic toxins, while harmful when in excess in the setting of kidney failure, might have other important “non-toxic” roles in normal biology, including metabolism, signaling, regulating redox state, and gut microbiome population dynamics. For example, there is evidence that indoxyl sulfate, at physiologic plasma concentrations, acts as a superoxide radical scavenger (39). Indoxyl sulfate can also cross the blood–brain barrier, where it may limit CNS inflammation in astrocytes and microglia (40). In mice, the oral administration of indole prevented the expression of key proteins in the NF-KB pathway and downstream inflammatory proinflammatory gene expression that followed the infusion of lipopolysaccharide (33). Importantly, several studies have described alterations in the composition of the microbiome in patients with advanced renal disease or ESRD. It now seems likely this “gut dysbiosis” reflects dietary alterations and an effect of uremic solutes on the composition of the gut microbiome (41).

If we consider uremia as a disturbance of remote sensing and signaling, we might say that CKD alters the normal functioning of a highly-regulated transporter and “drug” metabolizing enzyme network involved in sensing and signaling to maintain homeostasis of small organic molecules that have “high informational content” with respect to remote inter-organ and inter-organismal communication. The body's attempt to restore homeostasis in the face of the perturbations caused by uremic syndrome and ongoing progression of kidney disease then depends upon the homeostasis-promoting properties of several interacting systems: the remote sensing and signaling system, the neuroendocrine system, the growth factor-cytokine system, and the autonomic nervous systems.

## What Orchestrates the System?

If indoxyl sulfate is a key small molecule in a larger “remote sensing and signaling system” involved in inter-organ crosstalk within the body and inter-organismal communication mediated by transporters and “drug” metabolizing enzymes, it seems tempting to ask, what is the central conductor? For uremic toxins and uremic solutes derived from gut microbes, a plausible candidate for this role might be the gut microbiome–with its vast number of enzymes and signaling pathways. The small molecules produced by gut microbes, while perhaps of greatest current interest from the viewpoint of inter-organismal communication and kidney disease, do not, however, include the multitudes of endogenous metabolites and signaling molecules generated largely independent of the gut microbes and handled by “drug” transporters, “drug” metabolizing enzymes, and related proteins (42).

The Remote Sensing and Signaling Theory is a general theory that attempts to explain how multi-specific, oligo-specific, and monospecific transporters and enzymes work together, along with regulatory proteins (e.g., nuclear receptors, kinases) to achieve optimal levels of hundreds if not thousands of small molecules in different body tissues (e.g., CNS, kidney, liver, placenta) and body fluids [e.g., blood, CSF, urine, bile, amniotic fluid; (43–45)]. In such a multi-scale complex adaptive system, it is hard to posit a single orchestrator, for this is likely an emergent property of many interacting transporters, enzymes, nuclear receptors, metabolites, and signaling molecules—in many interacting organs and organisms.

While the systems pathophysiology of CKD is a new field, we conclude by again reminding the reader that the history of molecules related to indoxyl sulfate is very old. In the thirteenth century, Marco Polo described many caravans traveling from the Middle East and India that contained cloths dyed with the precious indigo. An important feature of indigo or indican was tight binding of the dye to cloth, and even in recent times, indigo serves as the dye that gives jeans their distinctive color. Once more we note that many such organic dyes played an important role in furthering our early understanding of pathology and physiology, including that involving protein-bound uremic solutes.

Here we have provided a glimpse of the physiological and pathophysiological history of a close relative of a highly valued dye employed to dye fabrics 6,000 years ago—from a solute found to accumulate in the serum of patients with renal disease, to a protein-bound uremic toxin, to a ligand of the renal “drug” transporters OAT1 and OAT3, to one of many signaling molecules participating in a “remote sensing and signaling system” that can now be conceptualized as a network of hundreds of proteins.

Viewing indoxyl sulfate and other uremic toxins through the multiscale systems biology perspective of the Remote Sensing and Signaling Theory suggests, as discussed elsewhere (33), a wide range of therapeutic possibilities to moderate the toxic effects of small protein-bound, gut-derived uremic toxins and uremic solutes. There is much more to come.

## Data Availability Statement

The original contributions presented in the study are included in the article/supplementary material, further inquiries can be directed to the corresponding author/s.

## Author Contributions

JL and SN contributed equally in the authorship of this submission. Both authors contributed to the article and approved the submitted version.

## Conflict of Interest

The authors declare that the research was conducted in the absence of any commercial or financial relationships that could be construed as a potential conflict of interest.
